# Diminished Telomeric 3′ Overhangs Are Associated with Telomere Dysfunction in Hoyeraal-Hreidarsson Syndrome

**DOI:** 10.1371/journal.pone.0005666

**Published:** 2009-05-22

**Authors:** Noa Lamm, Elly Ordan, Rotem Shponkin, Carmelit Richler, Memet Aker, Yehuda Tzfati

**Affiliations:** 1 Department of Genetics, The Silberman Institute of Life Sciences, The Hebrew University of Jerusalem, Givat Ram, Jerusalem, Israel; 2 Department of Pediatric Hematology-Oncology, Hadassah University Hospital, Ein Kerem, Jerusalem, Israel; 3 Department of Biochemistry, The Hebrew University-Hadassah Medical School, Ein Kerem, Jerusalem, Israel; Duke University, United States of America

## Abstract

**Background:**

Eukaryotic chromosomes end with telomeres, which in most organisms are composed of tandem DNA repeats associated with telomeric proteins. These DNA repeats are synthesized by the enzyme telomerase, whose activity in most human tissues is tightly regulated, leading to gradual telomere shortening with cell divisions. Shortening beyond a critical length causes telomere uncapping, manifested by the activation of a DNA damage response (DDR) and consequently cell cycle arrest. Thus, telomere length limits the number of cell divisions and provides a tumor-suppressing mechanism. However, not only telomere shortening, but also damaged telomere structure, can cause telomere uncapping. Dyskeratosis Congenita (DC) and its severe form Hoyeraal-Hreidarsson Syndrome (HHS) are genetic disorders mainly characterized by telomerase deficiency, accelerated telomere shortening, impaired cell proliferation, bone marrow failure, and immunodeficiency.

**Methodology/Principal Findings:**

We studied the telomere phenotypes in a family affected with HHS, in which the genes implicated in other cases of DC and HHS have been excluded, and telomerase expression and activity appears to be normal. Telomeres in blood leukocytes derived from the patients were severely short, but in primary fibroblasts they were normal in length. Nevertheless, a significant fraction of telomeres in these fibroblasts activated DDR, an indication of their uncapped state. In addition, the telomeric 3′ overhangs are diminished in blood cells and fibroblasts derived from the patients, consistent with a defect in telomere structure common to both cell types.

**Conclusions/Significance:**

Altogether, these results suggest that the primary defect in these patients lies in the telomere structure, rather than length. We postulate that this defect hinders the access of telomerase to telomeres, thus causing accelerated telomere shortening in blood cells that rely on telomerase to replenish their telomeres. In addition, it activates the DDR and impairs cell proliferation, even in cells with normal telomere length such as fibroblasts. This work demonstrates a telomere length-independent pathway that contributes to a telomere dysfunction disease.

## Introduction

Telomeres are the protective ends of eukaryotic chromosomes (reviewed in [1]–[3]). In most eukaryotes, telomeric DNA is composed of short tandem repeats and it ends with a single-strand 3′ overhang. In mammals, a complex of proteins named Shelterin binds the single- and double-stranded portions of the telomeres [1]. Telomeres shorten with each round of DNA replication, unless a specialized mechanism is present to compensate for this loss. In most eukaryotes, this compensation is carried out by the ribonucleoprotein (RNP) complex telomerase (reviewed in [4]). The telomerase catalytic core essentially comprises of an RNA moiety and a catalytic reverse transcriptase (in human, hTR and hTERT, respectively); hTERT copies a short template region within hTR onto the 3′ end of the telomere, thereby adding telomeric repeats. Telomerase activity and telomere length are regulated *in vivo* by additional telomerase subunits and by the Shelterin complex.

The 3′ overhang is a conserved and essential feature of the telomere [5]. This overhang, with the aid of the Shelterin proteins, invades an internal position within the telomere, forming a displacement-loop structure known as a T-loop [6]. The T-loop structure and the Shelterin complex protect the chromosome ends from nuclease degradation and suppress the DNA damage response (DDR), functions generally termed ‘telomere capping’ (reviewed in [1], [7], [8]). Telomere shortening past a critical length, shortening of the 3′ overhang, or damage to the T-loop structure or to the Shelterin complex all cause telomere uncapping, manifested by the activation of DDR, and cell-cycle arrest or apoptosis [9]–[12].

While hTR is constitutively expressed in all human cells, hTERT is barely expressed in somatic tissues. Even in highly proliferating cells such as stem cells and stimulated lymphocytes, which activate hTERT expression, the low levels of assembled telomerase RNP complexes are normally insufficient to maintain constant telomere length throughout life and only slow down the rate of shortening. In this way, telomere length sets a limit to the number of somatic cell divisions and provides a tumor-suppressing mechanism. Indeed, most cancer cells activate hTERT expression to high levels, which are sufficient to preserve constant telomere length and enable unlimited cell proliferation [13].

Dyskeratosis Congenita (DC) is a genetic disorder associated with accelerated telomere shortening (reviewed in [14]–[16]). Autosomal dominant, autosomal recessive, and X-linked forms of inheritance are known. DC has diverse clinical manifestations, including nail dystrophy, reticulate skin pigmentation, mucosal leukoplakia, and bone marrow failure (the main cause of mortality). Pulmonary fibrosis and high risk of cancer have also been associated with DC. Hoyeraal-Hreidarsson Syndrome (HHS) is a severe variant of DC. In addition to the typical DC symptoms, it is characterized by severe T^+^ B^−^ NK^−^ immunodeficiency (depletion of B lymphocytes and NK cells, combined with normal T lymphocytes counts), neurological developmental defects, and mortality at an early age [17]. DC and HHS are considered primarily telomerase-deficiency diseases, in which accelerated telomere shortening causes telomere uncapping and impaired cell proliferation. Indeed, tissues primarily exhibiting clinical symptoms are those with a high cell turnover, such as bone marrow and blood lymphocytes, which rely on telomerase to replenish their telomeres. Mutations in hTR, hTERT, and the box H/ACA proteins dyskerin, Nop10, and Nhp2 – all integral components of the telomerase RNP complex – were found in about half of the classical DC cases [18]–[22]. Recently, mutations in the gene encoding the Shelterin component Tin2 (*TINF2*) were also implicated in DC [23], [24]. In HHS, mostly mutations in the X-linked dyskerin gene (*DKC1*) were found [25], though a few other cases were reported with mutations in hTERT and *TINF2* [19], [24]. Nonetheless, the genetic defects in nearly half of DC and HHS patients are still unidentified. All the described mutations, except for those in Tin2, were shown to reduce the cellular level or activity of telomerase, and thus cause telomere shortening. Mutations in Tin2 are also associated with severe telomere shortening [24].

Recently, a pathogenic dyskerin mutation was reported to cause DC-like symptoms in mice [26]. Interestingly, although dyskerin is a telomerase component, this mutation induced DDR and impaired cell proliferation independent of telomere length. Together with the recent identification of Tin2 mutations [23], [24], [26], these findings suggest that failure to maintain telomere integrity in DC may arise by more than one pathway [15]. However, no specific telomere defect other than length has been reported in association with a human disease. In this report, we describe a family affected by HHS. We excluded mutations in the genes implicated so far in DC and HHS: *DKC1*, *hTERT*, *hTR, NOP10, NHP2,* and *TINF2*. We performed a detailed investigation of the telomeric phenotype in cells derived from the affected siblings and found no evidence for reduced level or activity of the telomerase catalytic core. Yet, telomeres in blood leukocytes were severely short, consistent with the clinical diagnosis of HHS. Surprisingly, telomeres in primary fibroblasts were normal in length. However, despite their normal length, they formed Telomere dysfunction-Induced Foci (TIFs), indicating a defect in the integrity of the telomere cap. In addition, the telomeric 3′ overhangs were found to be shortened in both blood leukocytes and fibroblasts derived from the patients. We therefore propose that the primary defect in this HHS-affected family is not telomere shortening but, rather, an impaired structure of the telomere.

## Results

### Clinical symptoms revealed a classical case of Hoyeraal-Hreidarsson syndrome

**Figure 1:**
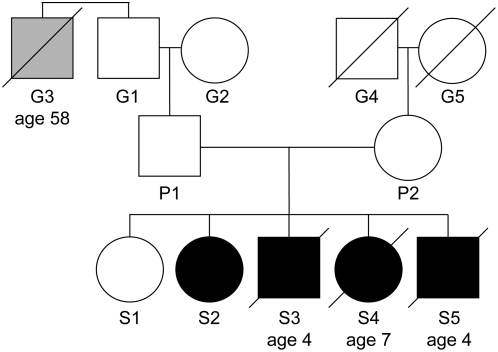
Genealogical tree of the HHS-affected family. Open circles and squares represent unaffected females and males, respectively. Black circles and squares represent affected females and males. A gray square indicates a family member who died from pulmonary fibrosis. Tilted lines indicate mortality, and the ages of mortality are indicated underneath.

The subject of this study was a family with five children, four of whom were diagnosed with HHS (Figure 1). The parents are healthy European descendents with no known consanguinity. The only known medical information that may be relevant is the death of the brother of the paternal grandfather from pulmonary fibrosis at the age of 58 (Figure 1: G3). The first sibling (S1), now 25 years old, is healthy and has no abnormal features. The diagnosis of the remaining siblings was based on typical clinical and laboratory features (Table 1), revealing the classical symptoms common to DC and HHS (nail dystrophy, leukoplakia, and bone marrow failure) and the typical HHS symptoms (severe T^+^ B^−^ NK^−^ immunodeficiency, intrauterine growth retardation, growth retardation, microcephaly, cerebellar hypoplasia, and esophageal dysfunction) [25]. Three of the affected siblings died at ages 3–7. S2, now 22 years old, underwent matched unrelated bone marrow transplantation, and currently (over three years post-transplantation) displays normal blood counts and normal immunological parameters.

**Table 1:** Clinical symptoms in the siblings of the HHS-affected family.

Symptoms\Siblings	S1	S2	S3	S4	S5
Skin pigmentation	−	+^1^	−	−	−
Nail dystrophy	−	+	+	+	+
Leukoplakia	−	+	+	+	+
Bone marrow failure	−	+	+	+	+
T^+^ B^−^ NK^−^ immunodeficiency	−	+	+	+	+
Intrauterine growth retardation	−	+	+	+	−
Growth retardation	−	−	+	+	+
Microcephaly	−	+	+	+	+
Cerebellar hypoplasia/dysfunction	−	+	+	+^2^	+^2^
Pancytopenia	−	+	+	+	+
Infections	−	+	+	+	+
Esophageal atresia	−	+	+^3^	+^3^	+^3^

1 Mild pigmentation was observed.

2 Cerebellar hypoplasia was confirmed by imaging.

3 History of major difficulties in swallowing solid food; not confirmed by imaging.

### Severely short telomeres in blood cells from the affected siblings

**Figure 2:**
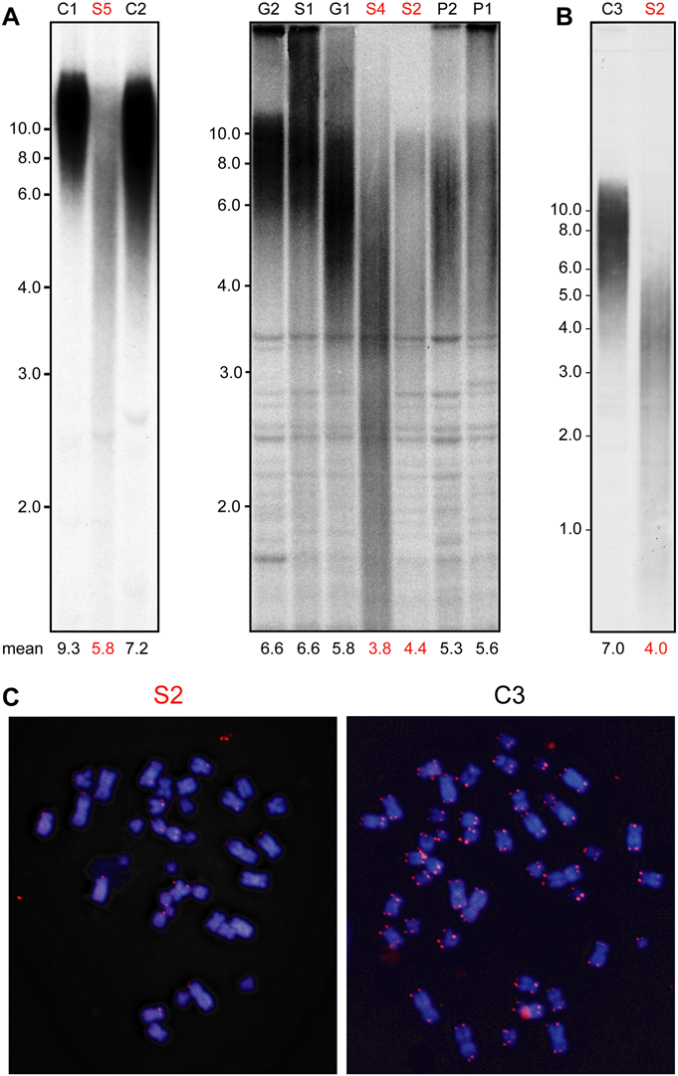
Severely short telomeres in the HHS patients. Genomic DNA was prepared from blood leukocytes (A) and EBV-infected lymphoblastoid cells (B), digested with the restriction endonuclease HinfI, and analyzed by Southern with a telomeric probe. The identity of the HHS-affected and unaffected individuals is indicated above the lanes by the same labels used in Figure 1. C1 and C2 indicate control samples taken from healthy unrelated individuals. The ages at which the blood samples were taken for telomere length analysis were (years): C1, 0.5; S5, 4; C2, 6; G2, 66; S1, 20; G1, 68; S4, 7; S2, 17; P2, 46; and P1, 46. The mean telomere length, calculated by the computer program *MATELO* [27], is indicated below the lanes. (C) S2 and C3 (control) LCLs were analyzed by FISH with a telomeric PNA probe. Both cultures grew for about the same population doublings and were processed side by side under the same conditions.

DC and HHS are characterized by severely short telomeres and impaired cell proliferation. We tested the average telomere length in peripheral blood leukocytes collected from the family members by Southern analysis of telomeric restriction fragments (Figure 2A). Telomeres of the affected siblings S2, S4, and S5 (there was insufficient S3 DNA for this analysis) were substantially shorter and showed a wider distribution of telomere lengths compared to those of the unaffected sibling S1, the paternal grandmother G2, and unrelated individuals C1 and C2 used as controls (mean telomere lengths of 4.4, 3.8, and 5.8 kb, compared to 6.6, 6.6, 9.3, and 7.2 kb, respectively, as calculated by the computer program *MATELO* [27]). Importantly, a significant portion of the S2, S4, and S5 telomeres were below 3 kb, considered short enough to trigger DDR and impair cell proliferation [3]. The telomeres of the parents P1 and P2 and the grandfather G1 were also relatively short (mean telomere lengths of 5.3, 5.6, and 5.8 kb, respectively) and had a wide distribution of lengths, although these individuals were healthy and bore no physical or laboratory abnormalities attributable to HHS. While such moderately short telomeres may fall within the variability in the population, they could, given the short telomeres of their HHS-affected children, indicate the genetic contribution of both parents to the disease. In particular, the telomeres of P1, instead of being longer than those of his parents (average telomere length in leukocytes is normally reduced with age), are comparable to those of his father (G1) and clearly shorter than those of his mother (G2), suggesting abnormal telomere shortening in the clinically unaffected P1. The relatively short telomeres of P1 and the case of pulmonary fibrosis in the paternal grandfather's family (Figure 1: G3) raised the possibility of paternal transmission of a mutation associated with the disease from G1 to P1 and to S2–5. We considered the possibility of paternal autosomal-dominant inheritance accompanied by anticipation – the aggravation of the disease phenotype in successive generations due to progressively shortening telomeres – as previously reported for DC [28]. According to this scenario, we expected the telomeres in sperm cells of the father (P1) to be shorter than normal. However, the telomeres in the sperm were comparable to those of the control (data not shown), indicating that the children did not inherit short telomeres from their father (P1). Although we cannot exclude maternal autosomal-dominant inheritance, the relatively short telomeres of both parents and the apparently normal telomere length in paternal sperm are consistent with recessive homozygous or compound heterozygous, rather than dominant heterozygous, inheritance.

The average telomere length of the parents (5.6 and 5.3 kb) was not dramatically longer than that of the affected siblings (4.4, 3.8, and 5.8 kb; Figure 2A). Therefore, average telomere length, in and of itself, cannot explain why the siblings were severely sick while the parents did not present any clinical signs attributable to telomere dysfunction (such as mild anemia or weakened immune response). Since a few critically short telomeres within a cell are sufficient to trigger DDR regardless of the average telomere length [29], it is possible that the severely short telomeres (below 3 kb) present in the affected siblings, but not in the healthy parents (Figure 2A) have a causal role in the disease. Another possible explanation for the difference between the healthy parents and the affected siblings is a telomere defect other than length, which contributes to telomere uncapping and to the pathology of the disease. Such a telomere defect in the affected siblings is suggested below.

We next examined whether a few telomeres in the affected cells are significantly shorter than others by telomere Fluorescent In Situ Hybridization (FISH) using a fluorescent peptide-nucleic acid (PNA) probe [30]. We used Epstein-Barr virus (EBV)-infected lymphoblastoid cell lines (LCLs) prepared from S2 and from an unrelated individual as a control. Both cultures were established using the same method and grown for approximately the same number of population doublings. The FISH procedure, microscope visualization, and image processing were performed side by side under the same conditions. The FISH signal corresponding to the telomeres was clearly weaker in the affected cells, as compared to the control (Figure 2C), indicating that most telomeres in the affected cells, and not just a small subset of the population, were significantly short. These results are consistent with a Southern analysis performed on the same cultures, revealing severely short telomeres (Figure 2B). Additional Southern analysis of several independent S2 and control LCLs at different population doublings showed that the telomeres in the S2 LCLs were severely short and continued to shorten with cell divisions. In contrast, the telomeres in the control LCLs were longer, and were stably maintained or even elongated with cell divisions (data not shown), as reported previously for LCLs in the pre-immortalized phase [31]. Although LCLs are not primary cultures and their telomere length does not necessarily represent the telomere length in the primary tissue from which they were derived, the continuous telomere shortening in the affected cells clearly indicates a defect in telomere length maintenance.

Short telomeres could potentially activate DNA repair mechanisms, resulting in telomere fusion, breakage, and chromosomal rearrangements. Indeed, high frequency of chromosomal aberrations have been previously reported in DC cases [32]. However, no such aberrations were observed in this case, whether by FISH of the S2 LCLs (Figure 2C) or by karyotype analysis of metaphase-chromosome spreads prepared from S2 and S4 primary lymphocytes (over 50 cells each; data not shown). We thus conclude that the disease is associated with short telomeres but apparently not with significant chromosomal instability.

### Proliferation of the HHS-affected cells in culture

**Figure 3:**
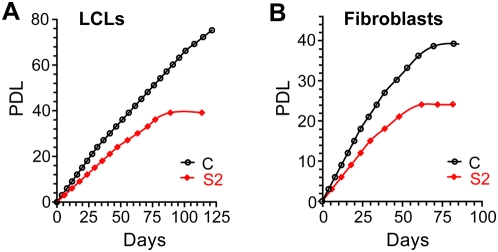
Impaired proliferation of cell cultures prepared from an HHS-affected patient. EBV-infected lymphoblastoid (A) and primary fibroblast (B) cultures were grown as described in Materials and Methods. The cumulative population doubling level (PDL) of the HHS-affected (S2) and control (C) cultures is drawn as a function of days in culture.

As described above and shown in Figure 2, severely short telomeres were found in S2, S4, and S5 blood leukocytes, and in S2 LCLs. Since short telomeres that hinder cell proliferation are believed to play a causal role in DC and HHS [33], we followed the growth rate of three S2 LCLs in culture. Indeed, these cultures grew two to three times slower than control cells (the growth of one culture is shown as an example in Figure 3A). Furthermore, while normal LCLs readily immortalize [31], the S2 LCLs stopped growing after about 40–50 population doublings, indicating their limited proliferative potential. We also examined the growth of S2 primary fibroblasts in culture. As reported previously for fibroblasts derived from DC patients [32], the S2 fibroblasts were larger, grew much slower, and stopped growing earlier than control fibroblasts (Figure 3B). Thus, impaired cell proliferation may have a major role in the pathology of the disease. In contrast, we observed normal proliferation of T lymphocytes taken from patients S2, S4, and S5 in response to the mitogens concanavalin A and phytohemagglutinin (data not shown), suggesting variable expression of the defect in different cell lineages. These results are consistent with the *in vivo* data showing T^+^ B^−^ NK^−^ type of immunodeficiency observed in these patients (Table 1), which is typical of HHS.

### The disease is not caused by a mutation in genes previously implicated in DC

Mutations causing DC were previously found in dyskerin, hTERT, hTR, Nop10, and recently also in Nhp2 and Tin2; of those, mutations in dyskerin, hTERT, and Tin2 were also found in HHS [18]–[25]. The presence of two affected girls and the random (normal) X inactivation pattern observed in blood cells taken from the mother (I. Dokal, personal communication) excluded the X-linked form of HHS and therefore a causal role for dyskerin. Genetic linkage analysis of the *hTR*, *hTERT*, *NOP10*, *NHP2,* and *TINF2* gene loci in the family members (P1–2, and S1–5) using microsatellite markers excluded the possibility that the siblings inherited a common mutation in these loci. This analysis also confirmed that the parents are not related. Additionally, *hTR*, *hTERT*, and *NOP10* were found to be normal in sequence (data not shown; and I. Dokal, personal communication). The Shelterin proteins Trf1, Trf2, Rap1, and Tpp1 were also excluded by linkage analysis. Finally, the linkage map of the *POT1* gene locus revealed the inheritance of the same paternal and maternal alleles by all the affected siblings but not by the unaffected one. However, we did not find a homozygous mutation in the POT1 open reading frame, nor any defect in the splicing pattern or expression level of the POT1 mRNA. We only identified a heterozygous single nucleotide polymorphism (SNP) in the paternal allele, which results in an amino acid change of Glycine to Valine at position 404 of POT1. This SNP has previously been identified in the population (dbSNP: rs35536751; average heterozygosity: 0.025), and while it is not likely to cause the disease on its own, it may still have a causal role by modifying the penetrance of another mutation. Altogether, our findings suggest that a homozygous or heterozygous disease-causing mutation is located in a gene or genes not yet known to cause DC or HHS. This mutation is yet to be identified.

### Normal hTR levels in the HHS-affected cells

**Figure 4:**
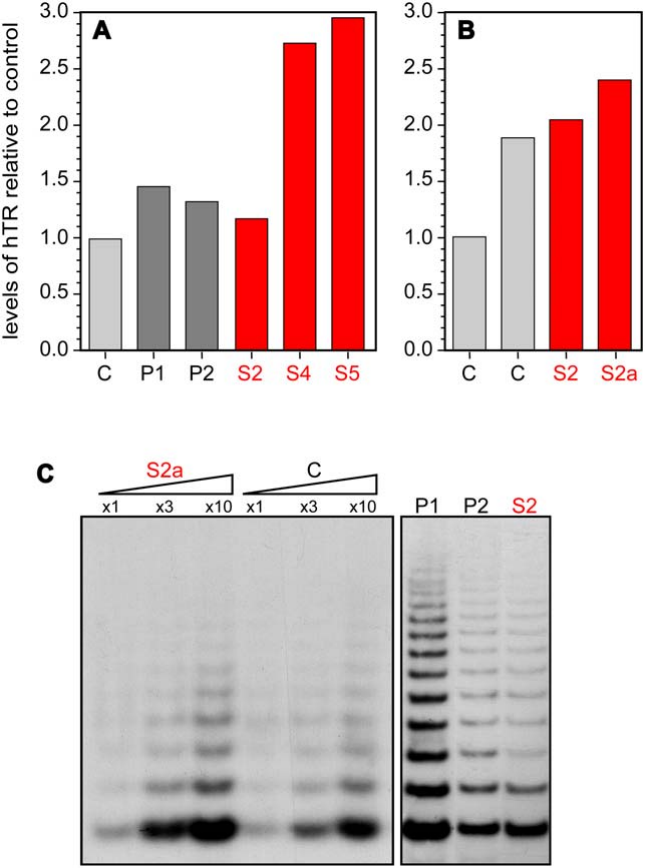
The HHS-affected cells express normal hTR levels and assemble active telomerase. Total RNA was prepared from blood leukocytes (A) and LCLs (B) derived from the affected siblings (red), unaffected parents (dark gray), or unaffected individuals (C – control; light gray). (A,B) hTR levels were measured by SYBR-green real-time RT-PCR, normalized to the levels of the U93 small nucleolar RNA, and presented as values relative to the controls. S2a indicates a later passage of the S2 LCL. Normalization to the levels of glyceraldehyde-3-phosphate dehydrogenase (GAPDH) mRNA did not significantly change the results (data not shown). (C) Whole-cell extracts were prepared from LCLs and assayed for telomerase activity by TRAP assay, as described in the Materials and Methods. The amounts of the extracts used in total protein were 10, 30, and 100 ng for the left panel, and 100 ng for the right panel. The PDL of the LCLs at which the samples were taken are: S2a, 38; C, 40; P1, 20; P2, 28; and S2, 25.

Mutations in dyskerin, Nop10, Nhp2, or the box H/ACA domain in hTR (the dyskerin binding site), result in reduced hTR levels and insufficient amount of assembled telomerase [21], [34], [35]. Although mutations in hTR, dyskerin, Nop10, and Nhp2 were excluded in the investigated family, it was still possible that a mutation in another unidentified gene impairs the expression or stability of hTR and thereby causes the disease. To test this possibility, we measured hTR levels in blood leukocytes and LCLs using real-time reverse-transcription (RT)-PCR. In the affected S2, S4, and S5 blood leukocytes and S2 LCLs we found hTR levels similar to, or up to 2.5-fold higher than, those measured in control cells (Figure 4A,B). These results exclude the possibility that reduced hTR levels cause the disease.

### The HHS-affected lymphoblastoid cells express hTERT and assemble active telomerase

Reduced hTR levels being excluded as described above, we investigated the ability of the affected cells to express hTERT and assemble a catalytically active telomerase. Unlike hTR, which is ubiquitously expressed in all human tissues, the expression of hTERT is restricted mostly to germ cells, stem cells, and stimulated lymphocytes. Following EBV infection, LCLs show fluctuations in hTERT expression until they immortalize and stably express relatively high levels of hTERT [31]. Therefore, a normal level of expression is difficult to define in the pre-immortalized phase. We measured the levels of hTERT mRNA in different passages of an S2 LCL by TaqMan real-time RT-PCR. The hTERT mRNA levels fluctuated between undetectable to relatively high levels of expression in a similar pattern to that observed for a pre-immortalized control LCL (data not shown), indicating that the S2 cells are capable of inducing hTERT expression at the mRNA level.

We next asked whether cultures expressing hTERT mRNA are also able to assemble active telomerase. We prepared cell extracts from the pre-immortalized LCLs and tested telomerase activity *in vitro* by the Telomeric Repeat Amplification Protocol (TRAP). The activity fluctuated between different passages of the same cultures, and between different cultures, both in the affected and in the control LCLs, correlating with the fluctuations in hTERT mRNA expression (Figure 4C and data not shown). LCLs expressing the hTERT gene, as indicated by the mRNA levels, were able to assemble a telomerase RNP complex that is active *in vitro*, and this ability was not compromised in the HHS-affected cells as compared to the control (Figure 4C; two different passages are shown for the S2 LCL). Importantly, despite the presence of active telomerase, the telomeres in S2 LCLs were severely short (Figure 2B,C) and continued to shorten until the cells stopped dividing (Figure 3A and data not shown). Since we did not observe any defect in the expression or *in vitro* activity of telomerase, we hypothesized that the defect in telomere maintenance in these cells lies not in the telomerase catalytic core, but rather in its recruitment or activation at the telomeres.

### Normal telomere length in the HHS-affected skin fibroblasts

**Figure 5:**
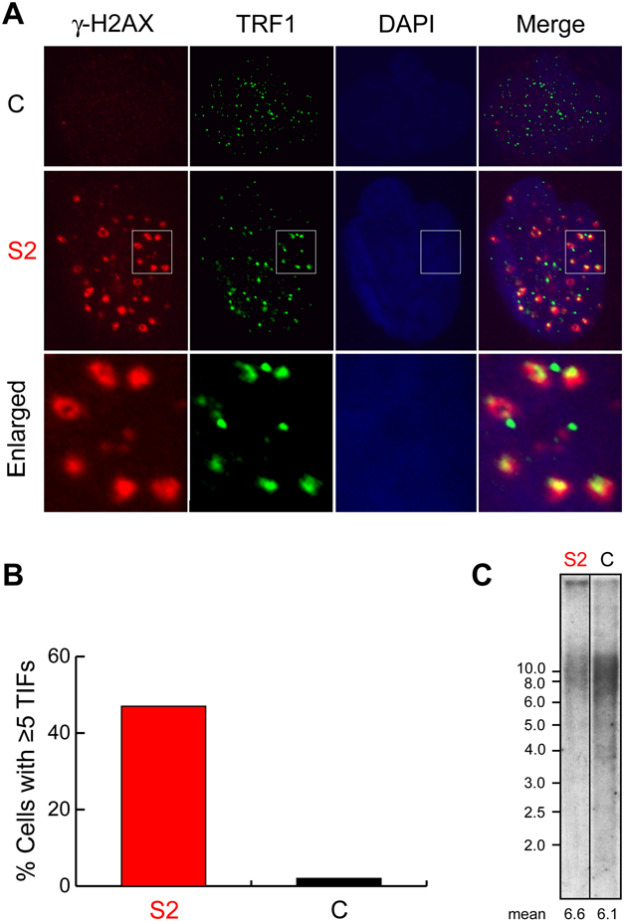
TIF formation in HHS-affected fibroblasts with normal telomere length. (A) Control (C) and HHS-affected (S2) primary fibroblast cultures (established at the ages of 30 and 17 years, and grown to PDL of 14 and 10, respectively) were immunostained for TRF1 (green) and γ-H2AX (red), and with DAPI for the nuclei (blue), as indicated above the images. The bottom panels show enlarged images that include several telomeres. The images of the affected and control cells were obtained and processed in the same way, side by side. (B) The number of TIFs (defined as colocalized TRF1 and γ-H2AX foci) was counted in randomly-chosen 67 affected and 58 control cells. The graph shows the percentage of cells with at least five such foci. (C) Genomic DNA was prepared from these cultures and the average length of telomeres estimated by Southern analysis.

**Figure 6:**
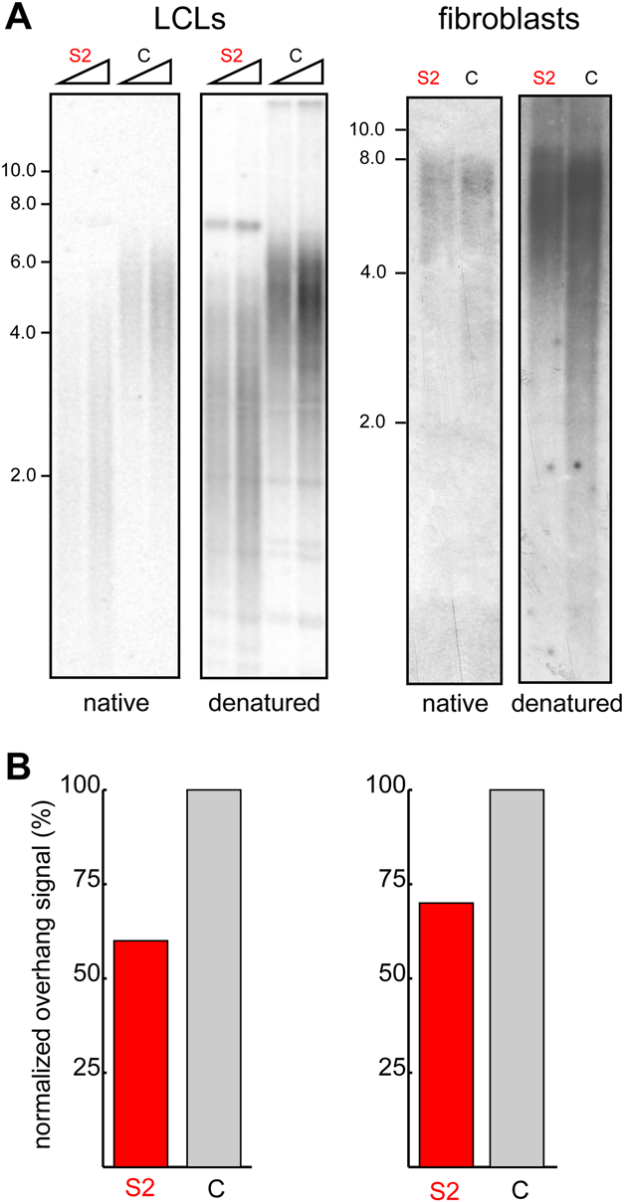
Reduced telomeric overhang signal in the HHS-affected cells. (A) Genomic DNA samples prepared from S2 and control LCLs (PDL of 44 and 50, respectively; 2 and 4 µg) or fibroblasts (PDL of 14 and 16 for S2 and C, respectively; 2 µg) were digested with MboI and AluI and electrophoresed in a 0.7% agarose gel. The average length of the 3′ overhang was estimated by in-gel hybridization of native DNA to a C-rich telomeric probe (native panels). The DNA was subsequently denatured *in situ* and re-hybridized to the same probe to measure the total TTAGGG repeat signal (denatured panels). (B) The histograms below the images represent the quantified native (overhang) signals normalized to the denatured (total) signals and presented as percentage of the normalized overhang signals of the controls.

Short telomeres are considered the primary defect and a hallmark of DC and HHS. Since telomeres in blood cells derived from the patients were significantly short (Figure 2), and since short telomeres had previously also been found in skin fibroblasts of DC patients [33], we initially assumed that primary fibroblasts derived from the HHS patients would also have short telomeres. Surprisingly, however, the average telomere length in S2 fibroblasts was comparable to that in the control cells (Figures 5C and 6A). Since both LCLs and primary skin fibroblasts showed impaired cell proliferation, these results point to a defect other than telomere length.

### Formation of TIFs in the HHS-affected skin fibroblasts

Uncapped telomeres form TIFs, where they associate with DDR factors such as the phosphorylated form of the histone H2AX (γ-H2AX) [12]. The formation of TIFs is considered an important step in telomere uncapping-induced senescence. If a telomeric defect causes telomere uncapping in the HHS-affected fibroblasts, we would expect the formation of TIFs in these cells. We searched for TIFs by co-immunostaining S2 fibroblasts for γ-H2AX and for the telomeric protein TRF1 [1]. Interestingly, about 40% of the S2 cells, as compared with 2% of the control cells, contained at least five γ-H2AX foci that colocalized with telomeres (Figure 5A,B). These results imply that a telomeric defect other than length elicits DDR in the affected fibroblasts, which in turn likely hinders cell proliferation through the activation of a DNA damage checkpoint and cell cycle arrest. TIF formation at normal-length telomeres has been observed upon genetic manipulations of Shelterin components such as POT1 and TRF2 [12], [36], or in mice with a pathogenic dyskerin mutation [26]; however, such a phenomena has not yet been reported in a human disease.

### Short telomeric overhangs in the HHS-affected cells

Dysfunctional telomeres caused by genetic manipulations in mouse and yeast were shown to be associated with abnormally long telomeric 3′ overhangs, generated by unregulated resection of the 5′ end [37], [38]. Shortening of the 3′ overhang has also been reported following POT1 knockdown in human cells and in association with replicative senescence caused by critically short telomeres in primary fibroblasts grown in culture [9], [11]. Since changes in the length of the telomeric 3′ overhang were associated with dysfunctional telomeres regardless of their overall length, we estimated the length of the overhang in S2 LCLs and fibroblasts by in-gel hybridization of a telomeric C-rich probe to native telomeres. The signal corresponding to the 3′ overhang (Figure 6A, native) was normalized to the overall telomeric hybridization signal after denaturation of the same gel (Figure 6A, denatured). We found that the overhang signal was decreased in the fibroblasts and LCLs by about 30% and 40%, respectively, suggesting a comparable decrease in the average overhang length (Figure 6B).

**Figure 7:**
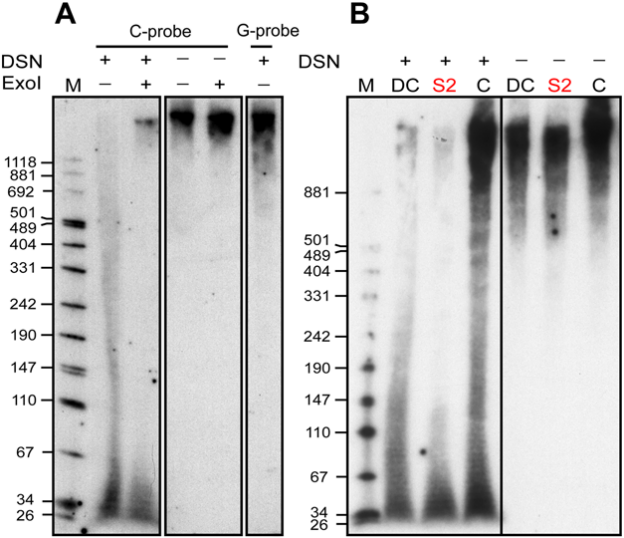
Diminished telomeric overhang length in the HHS-affected cells. (A) Four µg samples of genomic DNA prepared from control (unaffected) primary skin fibroblasts were treated with DSN, electrophoresed side by side in a denaturing 4–8% gradient polyacrylamide gel, and transferred onto a membrane, which was then cut and hybridized simultaneously with a G-rich and a C-rich probe. As controls we used one sample that was treated with 80 units of exonuclease I overnight prior to the DSN digestion (ExoI), and 60 ng samples of DNA, treated or untreated with exonuclease I but not with DSN. (B) Five µg samples of genomic DNA prepared from blood leukocytes taken from the HHS-affected patient (S2), unaffected individual (C), and an unrelated DC patient (DC) were treated with DSN, electrophoresed alongside untreated samples of 200 ng DNA, and blotted and hybridized with a C-rich probe as in (A).

To confirm this result and evaluate the distribution of overhang lengths (which is impossible by the in-gel method), we employed a recently published method that uses a duplex-specific nuclease (DSN) to digest the double stranded DNA [39]. The remaining single-stranded DNA is then separated by denaturing PAGE, blotted, and hybridized with a telomeric probe (Figure 7). In control leukocytes taken from a healthy person, we observed a wide distribution of hybridization signal with the C-rich probe, corresponding to lengths from 30 to above 1,000 nt. The signal corresponding to lengths of about 70–1000 nt disappeared upon the action of exonuclease I (Figure 7A), which specifically degrades single-stranded DNA in the 3′ to 5′ direction, confirming that the signal within this range represents telomeric 3′ overhangs. We ran a duplicated control sample digested with DSN and hybridized it with the G-rich probe simultaneously and under the same conditions as the C-rich probe hybridization (Figure 7A, G-rich lane). This control revealed the existence of high molecular weight DNA molecules with C-rich telomeric repeats. Since a signal in this size range was also observed upon digestion with exonuclease I and DSN and hybridization with the C-rich probe, we assume that some double-stranded telomeric DNA is resistant to DSN digestion and this signal does not represent telomeric overhangs. Interestingly, no hybridization signal with the G-rich probe was detected in the lower range, suggesting that the signal observed with the C-rich probe in the 30–70 nt range, although resistant to exonuclease I, does represent G-rich single-stranded telomeric sequence. It is possible that the 3′ ends in this fraction of overhangs are paired, for example in T-loop or quadruplex structures, and therefore are not available for exonuclease I digestion.

We examined the distribution of overhang lengths in three DNA samples extracted side by side from blood leukocytes using the same procedure. These samples were taken from the HHS patient S2, an unaffected control, and an unrelated DC patient. This DC patient was diagnosed at the age of 4 upon investigation of thrombocytopenia and mild anemia associated with moderate bone marrow hypoplasia. He displayed classical DC features and severely shortened telomeres in blood leukocytes (mean telomere length of 5.0 kb, as compared to 8.2 kb of his mother, calculated by Southern analysis of telomere restriction fragments using the computer program *MATELO* [27]; data not shown). The overhang signal in the HHS patient was dramatically diminished in the size range above 70 nt as compared to the unaffected individual, and barely detectable in the range above 150 nt (Figure 7B: compare S2 to C). Using the DSN method, we also observed diminished overhangs in fibroblasts with normal telomere length and S2 LCLs, and in S3 blood leukocytes, but not in leukocytes derived from the healthy sibling S1 (data not shown). Interestingly, the unrelated DC patient displayed a normal range of overhang lengths despite the dramatically short telomeres (Figure 7B: DC). Altogether, these results are consistent with the in-gel hybridization (Figure 6) and suggest that the diminished overhangs in the HHS patients are not the result of the severely short telomeres, but rather of another defect in telomere structure or composition that is specific to this case of HHS.

## Discussion

DC and its severe form, HHS, are inherited diseases associated with accelerated telomere shortening (reviewed in [14], [15], [40]). They have diverse clinical manifestations and variable age of onset. Mutations implicated in HHS were identified mostly in dyskerin, and recently also in hTERT and Tin2 [19], [24], [25]. Additional mutations in other telomerase subunits (Nop10, Nhp2, and hTR) were found in DC patients only [20]–[22]. With the exception of Tin2, these are integral components of the telomerase holoenzyme complex. Therefore, DC and HHS have been regarded primarily as telomerase-deficiency diseases, where insufficient compensation for telomere shortening impairs cell proliferation, and the disease manifests particularly in tissues with a high turnover [33]. The known cases of Tin2 mutations were also associated with severe telomere shortening [24] and so far there has been no report of a telomere defect other than length.

Since most of the known mutations causing HHS are in components of telomerase, we expected to find reduced levels or impaired *in vitro* activity of telomerase. Surprisingly, however, hTR levels in blood leukocytes and LCLs were normal or even elevated (Figure 4A), suggesting that hTR expression and stability, and probably also the box H/ACA protein complex, are not involved in the disease pathogenesis. The elevated hTR levels could be attributed to the activation of DDR at telomeres, as has previously been reported for UV-irradiated cells [41]. Moreover, the affected LCLs express hTERT and assemble telomerase that is active *in vitro* (Figure 4B,C). Hence, the ongoing telomere shortening in the presence of active telomerase in the affected LCLs, suggest that the cause for telomere shortening lies not in the telomerase catalytic core but rather in its recruitment or activation.

An interesting finding of our study is the variation in telomere length between different tissues taken from the same patient. While telomeres in blood leukocytes are significantly short (average length of 4.4 kb, Figure 2A), telomeres in fibroblasts appear normal in length (average length of 6.6 kb after growth in culture to PDL 10, Figure 5C). The formation of TIFs in these fibroblasts indicates the activation of DDR at telomeres, a typical consequence of telomere dysfunction [12]. Telomere capping has two main functions: the recruitment of telomerase and the suppression of DDR. In previously characterized DC and HHS cases, reduced telomerase activity resulted in accelerated telomere shortening to a critical length, which in turn activated DDR. In contrast, here we show that the activation of DDR in the HHS-affected primary fibroblasts is not caused by telomerase deficiency (because telomerase is not normally expressed in fibroblasts) or by short telomeres. Assuming the presence of the same telomere defect in blood leucocytes, it is plausible that telomere shorting is not the primary event causing the disease also in these cells. Since the major clinical symptoms were found in the hematopoietic system (Table 1), where short telomeres were observed, accelerated telomere shortening may still be the main direct cause of the clinical manifestation of the disease. We suggest that in the etiological pathway, a primary defect in telomere structure underlies the inability of the telomeres to both suppress DDR and to recruit and/or activate telomerase.

Another indication for a telomere defect that is independent of length was found in the telomeric 3′ overhang. Changes in the length of the telomeric overhang were shown to be associated with uncapping of normal-length telomeres achieved by genetic manipulation of Shelterin components [9], [37], [38]. However, changes in overhang length have not yet been reported in association with any human disease. Interestingly, we observed a reduction in the average 3′ overhang length in both LCLs and primary fibroblasts (Figure 6A,B). In addition, we used a newly developed method based on the degradation of double stranded DNA by a duplex-specific nuclease, leaving the overhang intact [39]. Using this method, we found a significant reduction in the 3′ overhang lengths in blood leukocytes, LCLs, and primary fibroblasts derived from the HHS patients, as compared with those measured for normal individuals (Figure 7 and data not shown). We next asked whether the diminished overhang length in blood leukocytes was a result of short telomeres. We obtained a blood sample from an unrelated DC patient and assayed the overall telomere length and the length of the overhangs. Interestingly, we found significantly longer overhangs in this DC patient as compared to the HHS patient (S2), although the overall telomere lengths were severely short in both patients (Figure 7B and data not shown). The telomeric overhang is the binding site for the POT1-TPP1 heterodimer, which is required for both telomerase action and DDR suppression [42]–[44]. It is tempting to speculate that the reduced overhang length impairs the ability of the POT1-TPP1 heterodimer to suppress DDR at the telomere ends. According to this hypothesis, it also impairs the ability of POT1-TPP1 to recruit or activate telomerase in cell types with high proliferation rates that express functional telomerase (such as cells of the hematopoietic system). This may explain the difference in telomere length between blood cells and fibroblasts, which is consistent with the more severe manifestation of the disease in the hematopoietic system than in the skin (Table 1).

Telomere shortening was suggested to be a ‘biological clock’ and a tumor-suppressing mechanism. DC and HHS have been considered primarily telomerase-deficiency diseases in which accelerated telomere shortening reduces the proliferative potential of cells. Here we report on a family diagnosed with HHS, in which impaired telomere structure, rather than telomerase deficiency, is the primary cause of the disease. We observed reduced telomeric overhang lengths in the affected cells, providing an insight into the molecular defect causing the disease in this family and possibly also in other DC and HHS patients. Taken together with the mutations found in telomerase subunits and in the Shelterin component Tin2, this work demonstrates that multiple pathways can contribute to a telomere dysfunction disease. Further mechanistic study of such cases, where telomere dysfunction is uncoupled from telomere length, will elucidate important implications of the telomere structure in telomere synthesis and cell proliferation.

## Materials and Methods

This study was approved by the Helsinki Committee for Human Studies of Hadassah University Hospital. Informed written consent was obtained from the participants in this study (or their parents in cases of minors).

### Cell culture

Dermal fibroblast cultures and EBV-infected lymphoblastoid cell lines (LCLs) were established in the Department of Human Genetics, Hadassah University Hospital, Ein Kerem, Jerusalem. Primary skin fibroblasts were grown in BIO-AMF-complete media or DMEM supplemented with 20% fetal calf serum, and LCLs were grown in RPMI supplemented with 20% fetal calf serum. All media and media supplements were purchased from Biological Industries, Beit Haemek, Israel.

### Microsatellite linkage analysis and DNA sequencing

Microsatellite linkage analysis, using the appropriate primers chosen from the complete human linkage mapping set (Applied Biosystems), and DNA sequencing, were done at the Center for Genomic Technologies, The Hebrew University of Jerusalem.

### Southern analysis of telomeric restriction fragments

Genomic DNA (2–5 µg) was digested overnight at 37°C with HinfI restriction endonuclease. Fragments were separated on a 0.7% agarose gel, transferred to a Hybond N+ membrane (GE Healthcare, life sciences), hybridized at 50°C with a telomeric oligonucleotide probe [5′ end-labeled (TTAGGG)_4_] according to Church and Gilbert [45], washed for 5 min twice with 0.2 M wash buffer (0.2 M Na_2_HPO_4_ pH 7.2, 1 mM EDTA, and 2% SDS) at room temperature and once with 0.1 M wash buffer at 50°C, and exposed to film. The mean telomere length was calculated by the computer program *MATELO* [27]

### Telomere FISH

Chromosome spreads of lymphoblastoid cells in metaphase were prepared, hybridized to a fluorescent PNA probe (composed of the C strand of three telomeric repeats and labeled with cy3) and stained with DAPI as described in [30], [46]. The chromosome spreads were visualized using a Zeiss axioplan II fluorescence microscope.

### *In vitro* telomerase activity

Cell extracts were prepared and analyzed for telomerase activity by the telomeric repeat amplification protocol (TRAP), modified from [47]. Briefly, cell extracts were prepared by the addition of 200 µl CHAPS buffer to a pellet of 1–2×10^7^ cells, incubation on ice for 30 min, and centrifugation at 14,000×rpm at 4°C for 30 min. The supernatant was stored at −70°C. Protein concentrations were determined by the Bradford method using protein assay reagent (Bio-Rad Laboratories). Cell extracts (10–100 ng total protein) were incubated with 0.1 µg of TS primer, dNTPs, and 1× TRAP reaction buffer, as described [47]. Extension was performed at 30°C for 1 h in a final volume of 47.5 µl. Then, 5 units of Taq polymerase (TaqZol; Tal-Ron Ltd, Rehovot, Israel), 0.2 µl of [α-^32^P]dCTP (10 mCi/ml, 3,000Ci/mMol), and 0.1 µg of the ACX primer in a volume of 2.5 µl were added, and 29 cycles of PCR amplification (30 sec at 94°C, 30 sec at 50°C, and 1 min at 72°C) were performed. Products were analyzed by electrophoresis in a native 12.5% polyacrylamide gel in 1× TBE, followed by gel drying and exposure to PhosphoImager or film.

### Indirect immunofluorescence (IF)

TIFs were visualized by IF of primary fibroblasts with anti-γ-H2AX and anti-TRF1 antibodies (kindly supplied by T. de Lange) as described in [9]. Slides were then visualized using a Zeiss Axioplan II fluorescence microscope.

### In-gel hybridization analysis of the telomeric 3′ overhang

In-gel G-overhang assay was performed as described [9]. Following electrophoresis, the Southern gel was dried at 50°C, prehybridized at 50°C for 1 h in 0.5 M Na_2_HPO_4_ pH 7.2, 1 mM EDTA, 7% SDS, and 1% BSA, and hybridized overnight at 50°C with a 5′ end-labeled (CCCTAA)_4_ oligonucleotide probe. After hybridization, the gel was washed three times with 4× SSC and once with 4× SSC and 0.1% SDS at 50°C (30 min each wash), and exposed to PhosphoImager or film. Following G-overhang hybridization, gels were denatured by incubation in 0.5 M NaOH and 1.5 M NaCl for 20 min, neutralized in 3 M NaCl and 0.5 M Tris–HCl pH 7.0 for 20 min, rinsed with H_2_O, prehybridized, hybridized, and washed as previously. To determine the relative overhang signal, the signal intensity for each lane was determined before and after denaturation using Image J software (http://rsb.info.nih.gov/ij/). The overhang signal was normalized to the signal after denaturation, and presented as percentage of the normalized overhang signal in the control cells.

### Telomeric 3′ overhang analysis by duplex-specific nuclease

The DSN reaction was performed as previously reported [39]. Briefly, 4–5 µg of genomic DNA were digested with 0.2 unit/µg DNA of DSN in a 20 µl reaction volume at 37°C for 60 minutes. The reaction was halted by adding 20 µl of formamide buffer (90% formamide, 1× TBE, bromophenol blue) and immediately transferred to 95°C for 5 min. The digested samples were resolved in duplicates (to enable C- and G-strand specific hybridization in parallel) on a gradient (4–8%) polyacrylamide gel containing 8 M urea in 1× TBE. The gel was semi-dry electroblotted in 0.5× TBE onto a Hybond N+ membrane (Amersham-GE), which was then split for separate hybridization with a C-rich [(AACCCT)_3_] or a G-rich [(AGGGTT)_3_] probe at 40°C, following Church and Gilbert [45]. The membrane was washed in 0.2 M wash buffer (0.2 M Na_2_HPO_4_ pH 7.2, 1 mM EDTA, and 2% SDS), twice at room temperature and once at 40°C (5 min each wash), and exposed to film.
